# Transcriptional Changes in Pearl Millet Leaves under Heat Stress

**DOI:** 10.3390/genes12111716

**Published:** 2021-10-28

**Authors:** Dejun Huang, Min Sun, Ailing Zhang, Jishan Chen, Jian Zhang, Chuang Lin, Huan Zhang, Xiaowen Lu, Xiaoshan Wang, Haidong Yan, Jianan Tang, Linkai Huang

**Affiliations:** 1 Herbivorous Livestock Research Institute, Chongqing Academy of Animal Sciences, Chongqing 402460, China; Xkyhdj@163.com (D.H.); cjshlj@163.com (J.C.); 2 Department of Grassland Science, Sichuan Agricultural University, Chengdu 611130, China; summin1028@163.com (M.S.); zhangal120@outlook.com (A.Z.); linchuang0022@163.com (C.L.); zhanghuanSichuan@outlook.com (H.Z.); luxiaowen1126@163.com (X.L.); wangqiqi_shan@126.com (X.W.); yanhd@vt.edu (H.Y.); 3 Sichuan Grassland General Work Station, Chengdu 610097, China; 4 Department of Horticulture, Virginia Tech, Blacksburg, VA 24061, USA

**Keywords:** heat stress, pearl millet, transcriptome sequencing, protein folding, ROS scavenging enzyme, flavonoid synthesis pathway

## Abstract

High-temperature stress negatively affects the growth and development of plants, and therefore threatens global agricultural safety. Cultivating stress-tolerant plants is the current objective of plant breeding programs. Pearl millet is a multi-purpose plant, commonly used as a forage but also an important food staple. This crop is very heat-resistant and has a higher net assimilation rate than corn under high-temperature stress. However, the response of heat resistant pearl millet has so far not been studied at the transcriptional level. In this study, transcriptome sequencing of pearl millet leaves exposed to different lengths of heat treatment (1 h, 48 h and 96 h) was conducted in order to investigate the molecular mechanisms of the heat stress response and to identify key genes related to heat stress. The results showed that the amount of heat stress-induced DEGs in leaves differs with the length of exposure to high temperatures. The highest value of DEGs (8286) was observed for the group exposed to heat stress for 96 h, while the other two treatments showed lower DEGs values of 4659 DEGs after 1 h exposure and 3981 DEGs after 48 h exposure to heat stress. The DEGs were mainly synthesized in protein folding pathways under high-temperature stress after 1 h exposure. Moreover, a large number of genes encoding ROS scavenging enzymes were activated under heat stress for 1 h and 48 h treatments. The flavonoid synthesis pathway of pearl millet was enriched after heat stress for 96 h. This study analyzed the transcription dynamics under short to long-term heat stress to provide a theoretical basis for the heat resistance response of pearl millet.

## 1. Introduction

High temperature is one of the stress factors often encountered in the growth cycle of plants. High temperature can cause degradation of heat-denatured abnormal proteins, damage the function of the cell membrane, cause plant cell damage or even cell death, thereby inhibiting plant growth and threatening food safety [1]. According to the IPCC (IPCC 2014; http://www.ipcc.ch/, accessed on 21 July 2021) report, the global average surface temperature from 2016 to 2035 will increase by 0.3–0.7 °C compared to the period from 1986 to 2005. Due to global warming, the production of millet and sorghum will decrease by 10–20% and 5–15%, respectively, with losses of 2.33–4.02 billion USD and 0.73–2.17 billion USD [2]. Furthermore, high temperature can lead to the death of several plant species, such as *Centaurea cyanus*, *Lilium brownii var*. viridulum Baker, *Mentha haplocalyx* [3]. Therefore, it is particularly important to identify key genes for heat resistance, understand their physiological role and use these genes to improve the heat resistance of crops and plants. Previous studies on plant heat stress mainly have two treatment methods [4]. Plants are exposed to heat stress 10–15 °C higher than the optimum growth temperature for a short time (heat shock treatment) [1], and plants are exposed to heat stress higher than the optimal growth temperature of 2.5 °C for a long time (prolonged warming) [5].

The production of pearl millet (*Pennisetum glaucum* (L.) R. Br) is the sixth largest cereal crop, right after wheat, rice, corn, sorghum and barley production [6]. Its planting area reaches 31 million hectares, which provides food and income sources of 90 million people (ICRISAT; https://www.icrisat.org/, 21 July 2021). Pearl millet has excellent tolerance to environmental stresses, especially high heat resistance, and can be planted in areas where other crops cannot survive [7]. The optimum growth temperature for its seedlings is 31 °C [8]. Therefore, studying the characteristics of heat-resistant crops such as pearl millet is helpful to understand abiotic stress response processes. Up to now, the research on pearl millet under heat stress is mainly conducted on phenotypic, physiological and molecular levels. Phenotypic physiology mainly focused on the germination rate [9], relative growth rate, net assimilation rate (NAR) [8], and peroxide scavenging enzyme activity [10] of pearl millet seeds under heat stress. At the molecular level, populations with stress-related genes, such as *SHSP* [11], *HAP 70* [12], *HSP 90* [13] and *PAP* [14] were mainly cloned. There were few reports on the transcription dynamics of pearl millet under heat stress, but the heat-resistance mechanisms of pearl millet are still unclear.

Based on the above background, we carried out heat stress experiments under controlled environment conditions using temperatures that are met under natural heat stress conditions (35–40 °C), and collected leaves after treatment for 1 h, 448 h and 96 h for transcriptome sequencing, in order to explore the transcriptional changes of pearl millet leaves under heat stress. This study lays the foundation for mining heat resistance-related genes in pearl millet and revealing the molecular mechanism of pearl millet response to heat stress.

## 2. Methods

### 2.1. Plant Material and Treatment

The seeds of pearl millet variety “Tifleaf 3” used in this research were provided by Sichuan Agricultural University, College of Animal Science and Technology, Department of Grassland, Chengdu, Sichuan, China. The pearl millet seeds were placed in a plastic box (10 × 15 × 6 cm) filled with quartz sand and cultivated in an artificial growth chamber at 26 °C under light for 14 h and at 21 °C in the dark for 10 h. The seedlings were cultured for 13 days and divided into a control group (CK) and a heat treatment group. The experimental conditions of the control group were set to 26 °C for 14 h under light and at 21 °C for 10 h in the dark. The conditions of the heat treatment group were 40 °C for 14 h under light and 10 h in the dark at 35 °C [6]. 50% Hoagland nutrition mix (1 mM MgSO_4_, 1 mM KH_2_PO_4_, 1 mM NH_4_NO_3_, 0.5 mM CaCl_2_, 0.1 mM FeNa-EDTA, 25 mM NaCl, 0.1 mM H_3_BO_3_, 0.1 mM Na_2_SiO_3_, 1.5 μM CuSO_4_, 50 μM KCl, 10 μM MnSO_4_, 0.075 μM Na_2_MoO_4_ and 2 μM ZnSO_4_) was used to provide nutrients throughout the cultivation stage and treatment period of the plant. The high-temperature treatment group started at the same time, and the changes of pearl millet seedlings were observed. It was found that there were differences in pearl millet seedlings after 48 h of heat stress, the leaves were sampled after 1 h, 48 h and 96 h according to the corresponding group (Figure S1). Leaves were placed in the cryogenic vials and immediately stored in an ultra-low temperature refrigerator at −80 °C, and then send the samples to Novogene (Tianjing, China) for paired-end sequencing. Three biological replicates were set up for each treatment, and a total of 18 samples were obtained.

### 2.2. RNA Extraction and cDNA Library Construction

RNA was extracted by RNeasy plant mini kit according to the instructions. The quality, purity and concentration of RNA were detected by NanoDrop spectrophotometer (Fremont, CA, USA) and Qubit 2.0 fluorometer system (Fremont, CA, USA). According to the specification, the cDNA library was constructed by NEBNext ^®^ Ultratm directional RNA library preparation kit. The mRNA was enriched by NEBNext Poly (A) mRNA Magnetic Isolation Module and cut into short fragments by Fragmentation Buffer. The cDNA strand was synthesized by random hexamer primers. After that, the second strand of cDNA was synthesized by adding buffer, dNTPs and DNA polymerase I. The double-strand cDNA was purified by AMPure XP beads. The purified cDNA was repaired, then the tail was added and sequenced. Finally, the cDNA library was obtained by PCR. Sequencing was performed using Illumina hi SEQ 2000.

### 2.3. Reads Mapping and Annotations

Software trimmatic (version 0.36) [15] was used to remove the original read adapter and low-quality nucleotide sequences. The filtered reads were detected by FastQC software (http://www.bioinformatics.babraham.ac.uk/projects/fastqc/, accessed on 26 June 2021) [16], and high-quality reads were used for downstream analysis. The Pacbio sequencing data of pearl millet (SRR 11816223) [17] published by previous researchers were used as the reference transcriptome. The Kallisto software was used to construct an index file (Kallisto index transcriptome.fa.gz -i transcriptome.indx), and then the gene expression level of each sample was quantified (Kallisto quant -i transcriptome.indx -b 100 -o outfile R1.fastq.gz R2.fastq.gez) [18]. Kallisto can directly compare the sequence of the RNA-Seq data to the transcriptome, which saves a lot of time. Finally, the differential expression analysis was completed by tximport [19] and DESeq [20] software. The genes with |log2 (heat treatment group/CK)| ≤ 1 and *p*.adjusted < 0.05 were identified as DEGs.

### 2.4. Gene Ontology, KEGG Ontology, Enrichment Analysis and Transcription Factor Identification

The functional identification of DEGs is based on the annotations of the pearl millet Pacbio sequencing (SRR 11816223) [17]. GO enrichment analysis was performed on DEGs using GOseq [21] R [22] software package, and the data set is the annotation of pearl millet Pacbio sequencing data (*p*.adjust < 0.05). The KEGG enrichment results were obtained by KOBAS 3.0 [23] analysis, and the data set is the annotation of pearl millet Pacbio sequencing data (*p* value < 0.05). Software iTAK [24] was used to identify transcription factors (TF).

### 2.5. K-Mean Analysis

K-means analysis was performed based on the Pearson correlation distance and the number of TPMs of DEGs. In order to avoid too many clusters, we set K = 15.

### 2.6. Determination of APX and SOD Activity

About 0.1 g of the weighed leaves (three biological replicates) were ground on the tissue grinder. After the tissue was broken, 1.5 mL of precooled 150 mM phosphate buffer was added, centrifuged at 12,000 rpm at 4 °C for 20 min, and the supernatant was collected as the crude enzyme extract. 

The SOD activity was measured by NBT (nitroblue tetrazolium) method [25]. First, 0.06 mM riboflavin, 195 mM L-methionine, 0.003 mM EDTA and 1.125 mM NBT were added to 50 μL of crude enzyme solution and reacted at 25 °C for 15–30 min (13,000 lux), and the color change of the solution was observed. Finally, when the color of the solution in the heat treatment group changed to bright purple and the color of the control group changed to dark purple, the reaction was stopped in the dark, and the absorbance value was measured at 560 nm.

The APX activity was measured according to Cakmak and Marschner [26]. 1.375mL of C_2_H_3_NaO_2_, 25 μL of EDTA, 25 μL of H_2_O_2_ and 25 μL of ascorbic acid in 50 μL of crude enzyme solution were added to 50 μL of crude enzyme solution. The absorbance was measured at 290 nm and recorded every 10 s. A total of 3 biological replicates were set up.

## 3. Results

### 3.1. High Quality of Transcriptome Data Obtained in Pearl Millet

Eighteen cDNA libraries (two treatments, three exposure types, three biological replicates) were sequenced through the Illumina Hi-Seq 2000 platform. A total of 590,796,912 original reads were obtained, and each library consisted of an average of 32,822,051 original reads. After filtering, 572,718,669 high-quality (HQ) reads were obtained, with an average of 31,817,703 reads per library. The average Q20 of the 18 cDNA libraries was 95.19%, the average Q30 was 92.66%, and the average GC content was 57.18% (Table S1). The correlation analysis between samples found that the Pearson index between the biological replicates was between 0.95–1, among which the correlation between CK_96hL2 (Processing method_point time L repeat) and CK_96hL5 was the lowest (0.95), and the correlation between H_1hL1 and H_1hL4 was the highest (Figure S2). The average pseudoalignment rate of 18 samples was 87.24%.

### 3.2. DEGs Related to Protein Folding and Photosynthesis Respond to Heat Stress in the Short and Medium Term, Respectively

**Figure 1:**
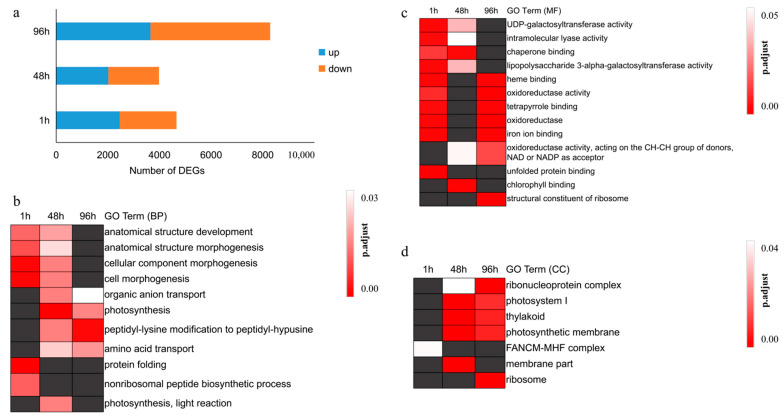
DEGs of pearl millet after 1 h, 48 h and 96 h heat stress. (**a**) Number of DEGs at 1 h, 48 h and 96 h of heat stress. (**b**) DEGs enrichment analysis at BP level. (**c**) DEGs enrichment analysis at MF level. (**d**) DEGs enrichment analysis at CC level. Gray represents not enriched.

Through software DESeq analysis, we identified 12,451 unique genes that were differentially expressed (Table S2). Among them 4659 DEGs were found after 1 h of heat stress exposure, 3981 DEGs after 48 h of heat stress exposure, and 8286 DEGs after 96 h of heat stress exposure (Figure 1a), which shows that long-term stress has a greater impact on the transcriptional level of pearl millet leaves. GO enrichment analysis was carried out on DEGs exposed to heat stress for 1 h, 48 h and 96 h (Figure 1, Table S3). Cell morphology (biological process, BP) and galactose-related transfer (molecular functions, MF) in the early and middle stages of heat treatment of pearl millet were the main processes of the response to the heat stress (Figure 1b,c). The DEGs related to photosynthesis (BP; cellular components, CC) and oxidoreductase activity of pearl millet were affected in the middle and long-term heat treatment (Figure 1b–d). Oxidoreductase (MF) and iron ion binding (MF) were the main enriched terms of DEGs in pearl millet after short-term and long-term heat treatment.

In addition to the shared GO terms mentioned above, there were significant differences in GO terms enrichment of DEGs at 1 h, 48 h and 96 h of heat stress. In the early stage of the heat stress (1 h), genes related to protein folding were rapidly enriched, such as protein folding” (BP) and “unfolded protein binding” (MF) to maintain protein homeostasis in the cell. DEGs in the group exposed to medium-term heat stress (48 h) were enriched in terms related to photosynthesis such as “photosynthetic electron transport chain” (BP), “chlorophyll binding” (MF) and “membrane part” (CC). This indicates that the photosynthesis of pearl millet was affected by the exposure to medium-term heat stress. The terms specifically enriched for DEG under long-term heat stress (96 h) at BP, MF, and CC levels were “peptide biosynthetic process”, “structural constituent of the ribosome”, and “ribosome” (Figure 1, Table S3).

### 3.3. Heat Stress Affects the Synthesis of Secondary Metabolites

**Figure 2:**
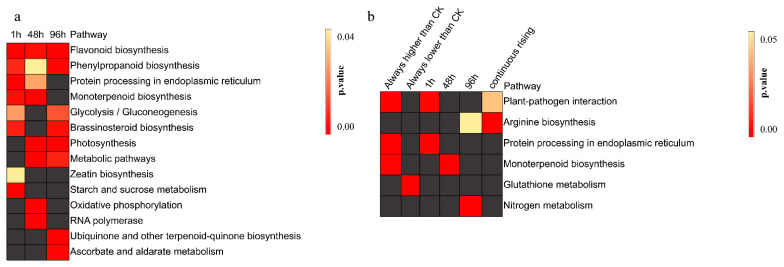
KEGG enrichment analysis of DEGs. (**a**) KEGG enrichment analysis of DEGs after 1 h, 48 h and 96 h heat stress. (**b**) KEGG enrichment analysis after K-means clustering. Gray represents not enriched.

KEGG enrichment showed that the short (1 h), medium (48 h), and long-term (96 h) DEGs under heat stress were enriched in the “Flavonoid biosynthesis” and “Phenylpropanoid biosynthesis” pathways (Figure 2a, Table S4). The above results demonstrated that pearl millet responds to heat stress by regulating the synthesis of secondary metabolites (flavonoids and phenylpropanoids). The genes related to “protein processing in endoplasmic reticulum pathway respond in the early and medium stages of heat stress, to avoid the destruction of protein homeostasis in pearl millet cells due to high temperature. With the extension of heat treatment time, genes related to “Photosynthesis” and “Metabolic pathways” in pearl millet leaves were negatively affected by high-temperature stress. We also found that the DEGs of pearl millet leaves were enriched in the “Glycolysis/Gluconeogenesis” and “Brassinosteroid biosynthesis” pathways after 1 h and 96 h of heat stress.

In addition to the above common pathways, there were processes that were specifically affected by heat stress at each time point. “Zeatin biosynthesis” and “Starch and sucrose metabolism” were pathways specifically enriched by DEGs under short-term heat stress (1 h). The DEGs of mid-stage heat stress (48 h) were mainly enriched specifically in the “Oxidative phosphorylation” and “RNA polymerase” pathways. DEGs were enriched in “ascorbate and aldarate metabolism” and “ubiquinone and other terpenoid quinone biosynthesis” pathways under long-term heat stress (96 h).

### 3.4. TF Families Affected by Heat Stress at Each Time Point

**Figure 3:**
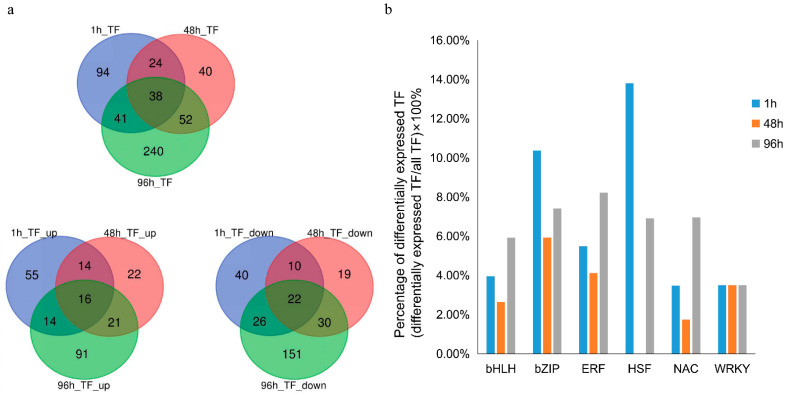
TF analysis of pearl millet under heat stress for1, 48, and 96 h. (**a**) The TFs Venn diagram of pearl millet showing the DEG overlaps after heat stress for 1, 48, and 96 h, including the Venn diagrams of all differentially expressed TFs, the up-regulated TFs Venn diagram and the down-regulated TFs Venn diagram. (**b**) The percentage of differentially expressed HSFs, bZIPs, ERFs, ARFs, NACs, wrkys and bhlhs (differential expression/total number).TF analysis of pearl millet under heat stress for 1, 48, and 96 h. Blue represents the differential expression percentage of TF in pearl millet after 1h of heat stress, orange represents the differential expression percentage of TF in pearl millet after 48 h of heat stress, and gray represents the percentage of differential expression of TF in pearl millet after 96 h of heat stress.

We identified a total of 529 differentially expressed TFs (transcription factors), distributed in 50 TF families. Differential expression of 197 TFs (99 up-regulated, 98 down-regulated) occurred after 1 h, 154 TFs (73 up-regulated, 81 down-regulated) occurred after 48 h and 371 TFs (142 up-regulated, 229 down-regulated) occurred after 98 h of heat stress. 117 TFs were differentially expressed at two-time points. A total of 16 differentially expressed TFs were up-regulated, and 22 differentially expressed TFs were down-regulated for all three exposure times (Figure 3). Among TFs related to stress response [27], HSF (44.83%) (differentially expressed HSF/total HSF) and bZIP (27.47%) (differentially expressed bZIP/total bZIP) accounted for the largest proportions after 1 h of stress (Figure 3b, Table 1). After 48 h of heat stress, bZIP (37.93%) and ERF (24.68%) were the most enriched, and after 96 h of heat stress, HSF (24.14%), NAC (11.85%) and bZIP (11.84%) were the most enriched (Figure 3b, Table 1). In conclusion, pearl millet activates specific TFs at different time points during heat.

### 3.5. K-Means Analysis Reveals That Protein Processing in the Endoplasmic Reticulum Is an Important Pathway for Pearl Millet to Respond to Heat Stress

**Figure 4:**
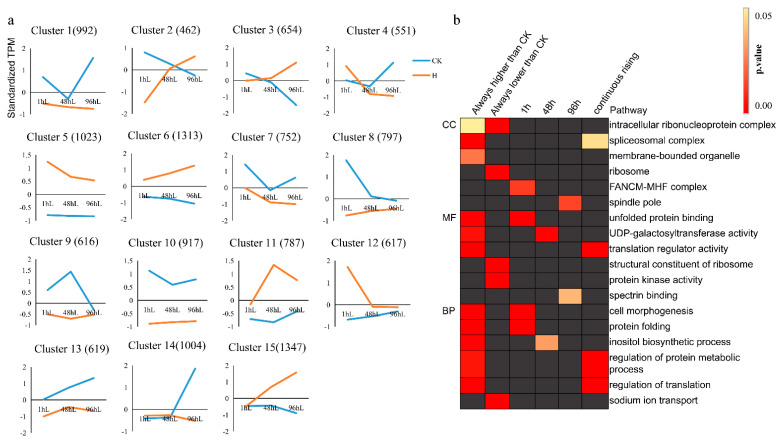
K-means analysis of DEGs. (**a**) Clustering of DEGs. (**b**) GO enrichment analysis after K-means clustering. The number in parentheses after Cluster indicates the number of DEGs. Gray represents not enriched.

The 12,452 DEGs were divided into 15 clusters by K-means. According to the overall trend, it can be divided into two groups: the expression level of the heat treatment group was always higher than CK and always lower than CK (Figure 4a). Under heat stress, the expression patterns of 5087 DEGs were higher than CK, and they belong to clusters 5, 6, 11, 12, and 15 respectively. There were 4074 DEGs that were always lower than CK, and they come from clusters 1, 7, 8, 9, and 10. KEGG enrichment analysis and GO enrichment analysis were performed on the above 5087 DEGs and 4074 DEGs respectively (Figure 2b and Figure 4b). The 5087 DEGs were enriched in the “spliceosomal complex” (CC), “membrane-bounded organelle” (CC level). At the MF and BP levels, 5087 DEGs were enriched in protein folding related, such as “unfolded protein binding” (MF) and “protein folding” (PB) (Table S5). The result of KEGG enrichment found that DEGs were enriched in a similar “Protein processing in endoplasmic reticulum” pathway (Table S6). The above results indicate that the regulatory network related to the maintenance of protein homeostasis in the cell was of irreplaceable importance in the response of pearl millet to heat stress. Glutathione plays an important role in plant response under stressful conditions [28]. However, in this study, DEGs with a lower expression pattern than CK were mainly enriched in the “glutathione metabolism” pathways. The 4074 DEGs were mainly enriched in “ribosome” and “intracellular ribonucleoprotein complex” terms at CC level, in “structural construct of ribosome” and “protein kinase activity” pathways at MF level, and in “sodium ion transport” and “photosynthetic electron transport chain” pathways at PB level.

The expression of DEGs in cluster 12 (total 617 DEGs), 11 (total of 787 DEGs) and 2 (total of 462 DEGs) was up-regulated after 1 h, 48 h and 96 h of heat stress, respectively. Enrichment analysis was performed on the three clusters. The GO enrichment results showed that cluster 12 was mainly enriched in the “FANCM-MHF complex” (CC), cluster 11 was not enriched at the CC level, and cluster 2 was mainly enriched in the “spindle pole” (CC). At the MF level, cluster 12 was mainly enriched in the “unfolded protein binding” pathway, cluster 11 was mainly enriched in the “UDP-galactosyltransferase activity” pathway, and cluster 2 was mainly enriched in the “spectrin binding” pathway. DEGs up-regulated at 1 h (cluster 12) were mainly enriched in “protein folding”, DEGs up-regulated at 48 h (cluster 11) were mainly enriched in “inositol biosynthetic process”, and DEGs up-regulated at 96 were mainly enriched in “regulation of protein metabolic process” (Table S5). DEGs in clusters 12, 11 and 2 were enriched in the “Protein processing in endoplasmic reticulum”, “Monoterpenoid biosynthesis” and “Nitrogen metabolism” pathways, respectively (Table S6).

In addition, we found that the expression patterns of DEGs in clusters 6 and 15 continued to rise under heat stress and contained a total of 2660 DEGs. The GO enrichment results of 2660 DEGs showed that “spliceosomal complex” (CC), “translation regulator activity” (MF) and “regulation of translation” (PB) were the most enriched pathways (Table S5). KEGG enrichment results demonstrated that 2660 DEGs were mainly enriched in the “Arginine biosynthesis” pathway (Table S6).

## 4. Discussion

Food security [29] caused by global temperature rise [30] and rapid population growth [31] are the main problems that human beings are facing. Pearl millet is an important cereal crop. Furthermore, it is rich in fermentable sugar, which might be a sustainable alternative energy source. In addition to that, pearl millet is known for its resistance to environmental adversity [32] and these valuable characteristics can help it to deal with the consequences of global warming [33]. Compared with maize, under high temperature stress (38/27 °C), the growth rate and net assimilation rate of pearl millet increased significantly [8]. However, there are only a few published studies focused on the effects of heat stress on pearl millet. Here, we performed RNA-sequencing on the leaves of pearl millet seedlings exposed to heat stress for 1 h, 48 h and 96 h, in order to discover the molecular mechanism of pearl millet response to short-term, medium-term and long-term heat stress. A total of 12,451 genes were differentially expressed in pearl millet at 1 h, 48 h and 96 h under heat stress. When pearl millet seedlings were subjected to heat stress, 4659 genes were involved in the short-term heat stress response process, the expression of 3981 genes was affected during medium-term heat stress, and 8286 DEGs play a role in the long-term heat stress. Transcription factor family analysis found that HSFs play a major role in the pearl millet response to short-term heat stress (Figure 3b, Table 1). During the medium-term heat stress, bZIP and ERF responded to stress, and NAC and bZIP mainly responded to long-term heat stress. This result indicated that the TF family response differs according to the length of stress exposure.

### 4.1. Rapid Removal of Misfolded Proteins in Cells Is an Important Pathway for Pearl Millet to Respond to Heat Stress

**Figure 5:**
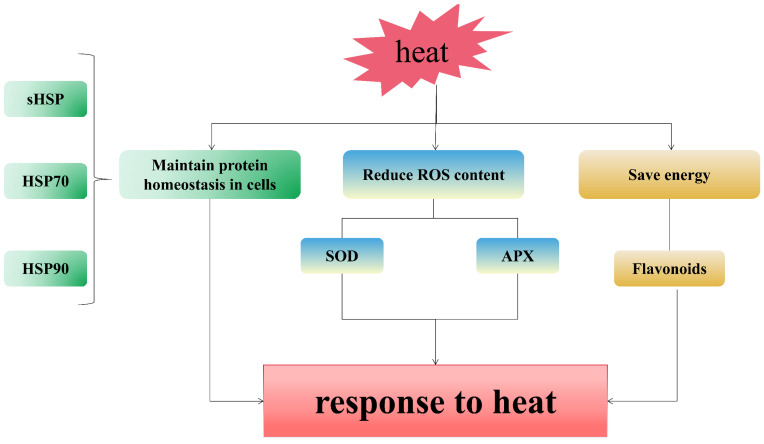
The response process changes in pearl millet with the passage of heat stress.

Our results clearly revealed that during the short-term heat stress, a large number of genes related to protein folding actively responded to heat stress (Figure 1, Table S2). Maintaining protein homeostasis in cells is another key process of plant response to stress. The HSPs family is an important member of the endoplasmic reticulum molecular chaperones. In 1987, Small heat shock protein (sHSP) was found in corn under heat stress [34]. In this study, two genes encoding sHSP were differentially expressed and both were up-regulated (Table 2). HSP70 [35] and HSP90 [36] can bind to unfolded peptide chains or denatured proteins to promote the correct folding or degradation of peptide chains and proteins, they play an important role in plant heat stress. In the early stage of heat stress in pearl millet, 25 *HSP70* were differentially expressed (88.00% were up-regulated) and 21 *HSP90* was differentially expressed (100.00% was up-regulated) (Table 2). HSP70 and HSP90 not only act as molecular chaperones, but they can also bind to HSFs and inhibit the activity of HSFs under normal conditions [37]. These results indicate that pearl millet quickly eliminates misfolded proteins caused by high temperature through HSPs, and maintains the stability of intracellular proteins, so that pearl millet can grow normally (Figure 5). We found that a large number of HSFs were up-regulated in pearl millet leaves under short-term heat stress (Table 1). This indicated that heat stress induced the production of a large number of unfolded or misfolded peptide chains, and HSP70/HSP90 bound to the peptide chains to release and activate HSFs.

### 4.2. Maintaining Stable ROS Content Is an Important Link for Pearl Millet to Respond to Short- and Mid-Term Heat Stress

Heat stress not only interferes with protein folding in plants, but also induces ROS production [37]. The increase of ROS content can cause lipid peroxidation of the cell membrane, protein inactivation, DNA damage, disrupt cell function, cause cell apoptosis and aggravate the impact of heat stress on plants [38]. However, ROS is not continuously produced [39]. ROS scavenging enzymes can remove ROS in plants, protect cells, and enhance plant tolerance under stress [37]. SOD is the first key enzyme in plant ROS scavenging enzymes, which transforms O_2_^−^ into H_2_O_2_ and oxygen [40]. In the early stage of heat stress (1 h), two differentially expressed *SOD* genes were up-regulated (Figure 5, Table 3). The generated H_2_O_2_ can enter the glutathione-ascorbate cycle and be cleared by APX [41]. APX is the most important ROS scavenger in plants [42]. When pearl millet was subjected to short-term and mid-term heat stress, 4 differentially expressed *APXs* were up-regulated (1 h), and 3 differentially expressed *APXs* were up-regulated (48 h). Under long-term heat stress, there were 2 differentially expressed *SOD* in pearl millet, one was up-regulated and the other one was down-regulated. Moreover, 4 differentially expressed genes encoding APX were all down-regulated. Physiological experiments have also confirmed that the activities of SOD and APX in pearl millet under short-term heat stress are higher than long-term heat stress (Figure S3). This indicated that compared with long-term heat stress, the ROS scavenging enzymes in pearl millet are more active in short-term heat stress, which is consistent with previous studies [17].

### 4.3. Pearl Millet Regulates the Synthesis of Secondary Metabolites in Order to Obtain More Energy in the Later Stage of Heat Stress

In recent years, a growing number of reports have shown that plants inhibit growth and development to maximize their viability under stressful conditions [43]. KEGG enrichment found that the DEGs of pearl millet leaves exposed to heat stress for 1 h, 48 h and 96 h were enriched in the “Flavonoid biosynthesis” pathways (Figure 2 and Figure 5, Table S3). Flavonoids are one of the secondary metabolites of plants. In addition to providing pigments for plant flowers, fruits, seeds and leaves [44], they also participate in the process of interaction between plants and the environment [45]. We tested the gene expression of the above pathways and we have identified 26 DEGs involved in the flavonoid biosynthesis pathway. They encoded C4H (cinnamate 4-hydroxylase), CHS (chalcone synthase) [46], CHI (chalcone isomerase) [47], F3′H (flavonoid 3′-hydroxylase), F3H (flavonoid 3-hydroxylase) [48], CYP75B1, HCT (shikimate O-hydroxycinnamoyltransferase) and caffeoyl-CoA O-methyltransferase, which are consistent with previous studies [49] (Table 4). Thus, we speculate that pearl millet inhibits flavonoid biosynthesis to save more energy to resist high temperature [50].

## 5. Conclusions

In order to explore the molecular mechanism of heat stress response of pearl, we performed transcriptome sequencing on the leaves of pearl millet after heat stress exposure of 1 h, 48 h and 96 h. In this study, a total of 12,451 DEGs were identified, the amount of DEGs increased with the higher exposure time to the heat stress which indicates that pearl reacts to the heat stress proportionally with the exposure time. We observed the expression of genes related to the maintenance of intracellular proteins in the early stage of heat stress, such as HSPs, which indicates that rapid correction and removal of misfolded proteins can effectively stabilize the intracellular protein homeostasis. In addition, a large number of genes encoding ROS scavenging enzymes are activated in the early and middle stages of heat stress to alleviate ROS damage caused by high temperature. Under stress, pearl millet regulates the synthesis of flavonoids and relieves the negative effects of heat stress. These findings provide a theoretical basis for the identification and exploitation of heat-resistant genes and lay a foundation for the study of heat-resistant mechanisms of pearl millet.

## Figures and Tables

**Table 1:** Expression of stress-related TF in pearl millet leaves under heat stress.

Annotation	ID	log2FC (H-1hL/CK-1hL)	log2FC (H-48hL/CK-48hL)	log2FC (H-96hL/CK-96hL)
ERF	i1_HQ_LWC_c16728/f8p0/1595	1.03	#N/A	#N/A
	i1_HQ_LWC_c2213/f2p0/1493	−1.38	#N/A	−2.55
	i1_HQ_LWC_c2324/f2p2/1682	#N/A	#N/A	−1.14
	i1_HQ_LWC_c8407/f5p2/1786	#N/A	#N/A	1.01
	i1_LQ_LWC_c11830/f1p1/1488	#N/A	#N/A	−2.25
	i1_LQ_LWC_c14109/f1p0/1383	−1.23	#N/A	−1.5
	i1_LQ_LWC_c20904/f1p47/1586	#N/A	#N/A	1.26
	i1_LQ_LWC_c21967/f1p3/1411	#N/A	#N/A	−1.83
	i1_LQ_LWC_c26384/f1p0/1341	#N/A	#N/A	−1.96
	i1_LQ_LWC_c36256/f1p0/1962	1.08	#N/A	#N/A
	i1_LQ_LWC_c37745/f1p24/1102	1.31	1.43	#N/A
	i1_LQ_LWC_c8826/f1p2/1547	#N/A	#N/A	−3.09
	i2_HQ_LWC_c37391/f2p0/2132	#N/A	#N/A	2.08
	i2_LQ_LWC_c105823/f1p0/2590	1.3	1.26	#N/A
	i2_LQ_LWC_c10690/f1p3/2523	#N/A	#N/A	−1.44
	i2_LQ_LWC_c12095/f1p2/2333	#N/A	−1.81	−1.63
	i2_LQ_LWC_c27703/f1p5/2133	#N/A	−3.16	−5.43
	i2_LQ_LWC_c37167/f1p2/3040	#N/A	#N/A	1.76
	i2_LQ_LWC_c48555/f1p0/2677	#N/A	#N/A	−1.94
	i2_LQ_LWC_c52587/f1p0/2111	#N/A	1.03	1.73
	i3_LQ_LWC_c20156/f1p0/3152	#N/A	#N/A	−1.5
	i3_LQ_LWC_c44933/f1p0/3022	#N/A	#N/A	1.07
bHLH	i0_LQ_LWC_c744/f1p0/869	#N/A	#N/A	−2.02
	i1_HQ_LWC_c16598/f3p0/1835	#N/A	#N/A	−1.87
	i1_HQ_LWC_c2277/f2p2/1974	#N/A	1.69	1.78
	i1_HQ_LWC_c2783/f5p0/1890	1.48	#N/A	#N/A
	i1_HQ_LWC_c3080/f2p1/1528	−1.07	#N/A	−1.09
	i1_LQ_LWC_c19586/f1p0/1685	#N/A	#N/A	−1.27
	i1_LQ_LWC_c21713/f1p0/1977	1.69	#N/A	#N/A
	i1_LQ_LWC_c26613/f1p0/1381	−2.61	#N/A	#N/A
	i1_LQ_LWC_c30650/f1p0/1843	1.3	#N/A	#N/A
	i1_LQ_LWC_c31312/f1p3/1421	#N/A	#N/A	4.17
	i1_LQ_LWC_c31916/f1p5/1737	#N/A	#N/A	−2.65
	i1_LQ_LWC_c32404/f1p0/1537	−1.07	#N/A	−1.77
	i1_LQ_LWC_c33040/f1p0/1932	1.8	#N/A	#N/A
	i1_LQ_LWC_c37910/f1p2/1185	−1.05	#N/A	−1.33
	i1_LQ_LWC_c3911/f1p3/1651	#N/A	#N/A	−2.88
	i1_LQ_LWC_c39446/f2p0/1411	#N/A	−1.49	−1.03
	i2_HQ_LWC_c34348/f2p3/2704	#N/A	2.03	2.51
	i2_HQ_LWC_c53137/f4p2/2329	#N/A	−1.43	−1.31
	i2_HQ_LWC_c7964/f3p0/2230	#N/A	#N/A	1.57
	i2_LQ_LWC_c106219/f1p1/2385	#N/A	#N/A	−2.2
	i2_LQ_LWC_c133064/f1p0/2039	#N/A	#N/A	−1.03
	i2_LQ_LWC_c36807/f1p4/2205	#N/A	1.53	#N/A
	i2_LQ_LWC_c47945/f1p0/2109	−3.23	#N/A	#N/A
	i2_LQ_LWC_c55090/f1p0/2091	#N/A	#N/A	1.56
	i2_LQ_LWC_c68318/f1p0/2330	#N/A	#N/A	1.39
	i2_LQ_LWC_c70175/f1p0/2208	−4.43	#N/A	#N/A
	i2_LQ_LWC_c8845/f1p0/2370	#N/A	#N/A	8.43
	i2_LQ_LWC_c9207/f1p0/2162	1.25	#N/A	1.07
	i2_LQ_LWC_c9259/f1p0/2884	1.33	1.78	1.47
bZIP	i0_LQ_LWC_c2149/f1p0/988	1.76	#N/A	#N/A
	i1_HQ_LWC_c11086/f2p0/1795	−1.26	#N/A	−1.57
	i1_HQ_LWC_c16237/f3p0/1669	1.25	#N/A	−1.21
	i1_HQ_LWC_c19844/f2p2/1556	#N/A	#N/A	−1.91
	i1_HQ_LWC_c2355/f7p4/1991	#N/A	#N/A	−1.85
	i1_HQ_LWC_c25566/f3p0/1372	#N/A	1.49	#N/A
	i1_HQ_LWC_c2716/f3p0/1731	1.52	#N/A	#N/A
	i1_HQ_LWC_c29681/f2p0/1990	1.6	#N/A	1.19
	i1_LQ_LWC_c12688/f1p0/1600	#N/A	#N/A	1.15
	i1_LQ_LWC_c16633/f2p0/1915	#N/A	−1.23	#N/A
	i1_LQ_LWC_c17140/f1p0/1733	#N/A	#N/A	−1.47
	i1_LQ_LWC_c17491/f1p0/1628	#N/A	#N/A	1.33
	i1_LQ_LWC_c18697/f1p0/1584	#N/A	#N/A	−3.62
	i1_LQ_LWC_c20333/f1p0/1549	#N/A	2.46	#N/A
	i1_LQ_LWC_c21844/f1p0/1626	#N/A	20.47	−21.21
	i1_LQ_LWC_c27131/f1p4/1187	1.31	2.52	1.8
	i1_LQ_LWC_c31593/f1p0/1765	−1.87	#N/A	−1.28
	i1_LQ_LWC_c3398/f1p5/1925	#N/A	#N/A	−1.08
	i1_LQ_LWC_c38399/f1p0/1812	#N/A	#N/A	−1.24
	i1_LQ_LWC_c6745/f1p1/1640	#N/A	#N/A	−1.33
	i1_LQ_LWC_c6814/f1p0/1757	#N/A	2.1	#N/A
	i2_HQ_LWC_c17257/f2p0/2441	#N/A	1.43	1.53
	i2_HQ_LWC_c4682/f2p1/2509	1.96	#N/A	#N/A
	i2_HQ_LWC_c5745/f2p2/2371	−5.28	−4.07	−4.77
	i2_LQ_LWC_c10506/f1p0/2572	1.12	#N/A	#N/A
	i2_LQ_LWC_c106423/f1p0/2422	1.4	1.15	#N/A
	i2_LQ_LWC_c114370/f1p0/2103	#N/A	#N/A	−1.26
	i2_LQ_LWC_c13727/f1p3/2077	1.28	#N/A	1.66
	i2_LQ_LWC_c24408/f1p0/2193	1.41	#N/A	1.24
	i2_LQ_LWC_c24803/f1p2/2716	−2.06	−2.37	−7.89
	i2_LQ_LWC_c25536/f1p2/2931	−2.65	−3.23	−2.11
	i2_LQ_LWC_c41884/f1p1/2042	#N/A	#N/A	−1.1
	i2_LQ_LWC_c46944/f1p7/2377	#N/A	#N/A	−1.7
	i2_LQ_LWC_c49205/f1p1/2747	#N/A	#N/A	1.85
	i2_LQ_LWC_c52220/f1p0/2449	2.74	#N/A	#N/A
	i2_LQ_LWC_c70730/f1p2/2959	−4.38	−6.06	#N/A
	i2_LQ_LWC_c77190/f1p4/2024	#N/A	#N/A	−2.62
	i2_LQ_LWC_c81747/f2p2/2111	#N/A	#N/A	7.92
	i2_LQ_LWC_c82592/f1p1/2865	#N/A	−1.02	−1.5
	i3_LQ_LWC_c21418/f1p0/3155	1.15	1.98	2.09
	i3_LQ_LWC_c36882/f1p6/3993	1.56	#N/A	#N/A
	i3_LQ_LWC_c40088/f1p9/3066	−4.82	−2.41	−3.43
	i4_HQ_LWC_c2296/f2p3/4925	−4.23	−2.52	−2.52
	i4_LQ_LWC_c22019/f1p0/4881	−3.24	−2.56	−2.21
	i5_LQ_LWC_c10548/f1p12/5049	1.57	#N/A	#N/A
	i7_LQ_LWC_c161/f1p0/7959	#N/A	#N/A	−5.05
HSF	i1_LQ_LWC_c18736/f1p1/1715	2.58	#N/A	#N/A
	i1_LQ_LWC_c22025/f1p2/1544	#N/A	#N/A	−1.93
	i2_HQ_LWC_c2225/f5p1/2358	#N/A	#N/A	−4.23
	i2_HQ_LWC_c3339/f2p3/2212	1.4	#N/A	2.09
	i2_HQ_LWC_c63561/f5p1/2436	#N/A	−1.62	−3.11
	i2_HQ_LWC_c7535/f4p0/2304	#N/A	#N/A	−2.3
	i2_LQ_LWC_c4082/f1p0/2693	1.01	#N/A	1.13
	i2_LQ_LWC_c49668/f1p0/2181	#N/A	#N/A	−2.36
	i2_LQ_LWC_c86456/f1p1/2142	#N/A	#N/A	−3.2
	i2_LQ_LWC_c90147/f1p0/2328	3.71	#N/A	#N/A
	i3_LQ_LWC_c14336/f1p0/3022	#N/A	#N/A	−2.76
NAC	i1_HQ_LWC_c1312/f6p0/1522	#N/A	#N/A	−2.02
	i1_HQ_LWC_c29731/f2p0/1677	#N/A	#N/A	−1.41
	i1_HQ_LWC_c8531/f2p0/2019	#N/A	#N/A	−1.44
	i1_LQ_LWC_c11267/f1p0/1925	−1.15	#N/A	#N/A
	i1_LQ_LWC_c12732/f1p0/1978	#N/A	#N/A	2.52
	i1_LQ_LWC_c23303/f1p0/1691	#N/A	#N/A	−3.57
	i1_LQ_LWC_c24307/f1p5/1514	#N/A	−1.18	−1.35
	i1_LQ_LWC_c31004/f1p0/1806	#N/A	#N/A	1.11
	i1_LQ_LWC_c32800/f1p2/1730	−1.03	#N/A	−1.92
	i1_LQ_LWC_c33678/f1p1/1854	#N/A	#N/A	−3.12
	i1_LQ_LWC_c36521/f1p0/1791	1.27	1.39	#N/A
	i1_LQ_LWC_c37681/f1p0/1237	−1.66	#N/A	−1.52
	i1_LQ_LWC_c5458/f1p0/1856	1.34	#N/A	#N/A
	i1_LQ_LWC_c6109/f1p2/1914	−2.38	#N/A	−2.31
	i1_LQ_LWC_c6781/f1p0/1635	#N/A	−1.65	#N/A
	i1_LQ_LWC_c9680/f1p3/1862	#N/A	#N/A	−3.58
	i2_HQ_LWC_c100859/f2p3/2422	2.79	#N/A	#N/A
	i2_HQ_LWC_c16790/f4p2/2632	1.03	#N/A	#N/A
	i2_HQ_LWC_c2298/f8p3/2112	#N/A	1.1	#N/A
	i2_HQ_LWC_c78151/f10p2/2488	#N/A	#N/A	2.67
	i2_LQ_LWC_c104913/f1p2/2561	#N/A	#N/A	2.33
	i2_LQ_LWC_c105082/f1p0/2164	#N/A	#N/A	−3.66
	i2_LQ_LWC_c105085/f1p1/2429	#N/A	#N/A	−1.29
	i2_LQ_LWC_c106176/f1p1/2359	#N/A	#N/A	1.52
	i2_LQ_LWC_c106220/f1p0/2782	#N/A	#N/A	−3.88
	i2_LQ_LWC_c115202/f1p2/2027	−1.28	−1.06	#N/A
	i2_LQ_LWC_c6544/f1p1/2222	#N/A	#N/A	−1.42
	i2_LQ_LWC_c90026/f1p1/2514	#N/A	#N/A	1.16
	i3_LQ_LWC_c12481/f1p0/3622	#N/A	#N/A	2.13
	i3_LQ_LWC_c13300/f1p0/3336	#N/A	#N/A	−7.52
	i4_LQ_LWC_c14651/f1p0/4901	#N/A	#N/A	3.69
WRKY	i1_HQ_LWC_c24104/f2p8/1933	#N/A	#N/A	−1.57
	i1_LQ_LWC_c10658/f1p1/1781	#N/A	#N/A	−4.64
	i1_LQ_LWC_c21040/f1p2/1452	#N/A	#N/A	−3.08
	i1_LQ_LWC_c35088/f1p0/1805	#N/A	#N/A	3.06
	i1_LQ_LWC_c36127/f1p0/1881	#N/A	−1.28	−1.11
	i1_LQ_LWC_c36450/f1p2/1858	1.64	1.54	1.35
	i2_LQ_LWC_c13758/f1p0/2102	#N/A	#N/A	−2.59
	i2_LQ_LWC_c3413/f1p0/2222	#N/A	21.72	#N/A
	i2_LQ_LWC_c8508/f1p0/2662	−1.08	#N/A	−1.58
	i2_LQ_LWC_c85901/f1p0/2168	1.62	#N/A	#N/A
	i3_LQ_LWC_c36452/f1p28/3212	1.75	1.6	1.63
	i7_LQ_LWC_c306/f1p0/7354	#N/A	#N/A	−1.82

**Table 2:** Expression of Protein Folding Related Genes in Pearl Millet Leaves under Heat Stress for 1 h.

Annotation	ID	log2FC (H-1hL/CK-1hL)	log2FC (H-48hL/CK-48hL)	log2FC (H-96hL/CK-96hL)
sHSP	i0_HQ_LWC_c184/f2p0/791	6.34	4.00	2.39
	i0_LQ_LWC_c967/f1p0/765	8.77	6.55	5.04
HSP70	i2_LQ_LWC_c35762/f1p8/2557	2.38	#N/A	#N/A
	i2_LQ_LWC_c82615/f1p8/2280	3.81	1.81	1.80
	i2_LQ_LWC_c105136/f1p0/2340	9.62	6.07	4.35
	i2_LQ_LWC_c105813/f1p8/2359	3.76	#N/A	2.42
	i2_LQ_LWC_c106010/f1p8/2392	3.82	1.79	2.17
	i2_LQ_LWC_c102949/f1p3/2116	7.95	6.25	#N/A
	i2_LQ_LWC_c109050/f1p8/2511	3.46	1.70	1.67
	i2_HQ_LWC_c43630/f6p12/2432	7.21	5.62	5.56
	i2_HQ_LWC_c50321/f2p2/2517	1.58	1.38	1.10
	i2_HQ_LWC_c69799/f2p1/2652	2.17	#N/A	1.06
	i2_HQ_LWC_c127192/f35p1/2517	1.94	#N/A	#N/A
	i1_LQ_LWC_c32521/f1p1/1976	1.90	1.79	#N/A
	i2_LQ_LWC_c4855/f1p10/3010	3.34	1.78	1.59
	i2_LQ_LWC_c7394/f1p7/2310	−1.45	#N/A	−1.66
	i2_LQ_LWC_c3333/f1p2/2529	5.10	3.47	2.62
	i2_LQ_LWC_c22856/f1p14/2305	1.11	1.43	1.04
	i2_LQ_LWC_c11597/f1p0/2377	2.60	#N/A	#N/A
	i2_LQ_LWC_c50733/f1p0/2426	2.94	2.67	1.87
	i2_LQ_LWC_c48249/f1p5/2266	−1.34	#N/A	−1.42
	i2_LQ_LWC_c53787/f1p5/2383	3.47	4.62	#N/A
	i2_LQ_LWC_c66032/f1p53/2457	1.45	#N/A	−1.87
	i2_LQ_LWC_c81928/f1p8/2468	2.65	#N/A	1.88
	i2_LQ_LWC_c82227/f1p0/2839	−4.52	#N/A	#N/A
	i2_LQ_LWC_c104760/f1p1/2446	2.07	1.82	1.11
	i3_LQ_LWC_c27490/f1p0/3150	2.98	3.35	3.12
HSP90	i2_LQ_LWC_c8803/f1p15/2771	1.90	#N/A	−1.85
	i2_LQ_LWC_c33469/f1p1/2516	2.61	#N/A	#N/A
	i2_LQ_LWC_c90259/f1p14/2485	1.84	#N/A	#N/A
	i2_LQ_LWC_c104439/f1p16/2591	2.25	#N/A	#N/A
	i2_LQ_LWC_c121080/f1p8/2511	10.70	10.13	9.17
	i2_LQ_LWC_c126787/f141p12/2519	1.53	#N/A	#N/A
	i3_LQ_LWC_c19538/f1p2/3645	7.78	7.27	6.06
	i4_LQ_LWC_c19908/f1p0/5045	2.36	#N/A	#N/A
	i2_HQ_LWC_c47794/f3p1/2946	4.84	2.72	1.69
	i2_HQ_LWC_c49563/f2p1/2825	4.64	3.59	3.33
	i2_HQ_LWC_c126707/f9p17/2641	3.19	2.01	2.81
	i2_HQ_LWC_c127285/f2p25/2794	3.36	#N/A	#N/A
	i1_LQ_LWC_c22321/f1p0/1789	5.38	#N/A	4.94
	i2_LQ_LWC_c3878/f1p2/2975	2.08	2.12	1.50
	i2_LQ_LWC_c25419/f1p24/2880	2.65	2.14	1.94
	i2_LQ_LWC_c33264/f1p2/2930	1.64	2.29	2.48
	i2_LQ_LWC_c35607/f1p2/2963	2.27	2.30	1.83
	i2_LQ_LWC_c42955/f2p2/2905	1.89	2.09	1.32
	i2_LQ_LWC_c82169/f1p11/2786	3.20	1.45	#N/A
	i2_LQ_LWC_c106886/f1p1/2786	4.84	3.57	2.97
	i6_LQ_LWC_c1262/f1p0/6420	2.94	#N/A	2.71

**Table 3:** Expression of ROS scavenging enzymes in pearl millet under heat stress for 1 h and 48 h.

Annotation	ID	log2FC (H-1hL/CK-1hL)	log2FC (H-48hL/CK-48hL)	log2FC (H-96hL/CK-96hL)
SOD	i0_LQ_LWC_c2218/f1p0/833	1.51	#N/A	#N/A
	i0_LQ_LWC_c429/f1p0/846	2.56	1.81	1.20
APX	i1_HQ_LWC_c40231/f3p0/1461	#N/A	1.03	#N/A
	i1_LQ_LWC_c18173/f1p0/1818	1.47	#N/A	−2.29
	i1_LQ_LWC_c18498/f1p3/1627	#N/A	1.30	#N/A
	i1_LQ_LWC_c38632/f1p0/1092	1.65	1.41	#N/A
	i2_LQ_LWC_c66599/f1p1/2322	1.12	#N/A	−1.74
	i6_LQ_LWC_c640/f1p0/6667	2.10	#N/A	#N/A

**Table 4:** Expression of Flavonoid Biosynthesis Related Genes in Pearl Millet Leaves under Heat Stress for 96 h.

Annotation	ID	log2FC (H-1hL/CK-1hL)	log2FC (H-48hL/CK-48hL)	log2FC (H-96hL/CK-96hL)
C4H	i1_HQ_LWC_c27582/f8p0/1861	#N/A	#N/A	−1.54
	i1_HQ_LWC_c10429/f5p1/1861	#N/A	#N/A	−1.56
	i1_LQ_LWC_c7551/f1p5/1917	#N/A	#N/A	−4.07
CHS	i1_HQ_LWC_c1494/f3p0/1849	−3.63	#N/A	−2.63
	i1_LQ_LWC_c3272/f1p0/1605	#N/A	−3.62	−7.70
	i1_LQ_LWC_c33680/f1p2/1647	−4.07	−2.25	−3.29
	i2_LQ_LWC_c18273/f1p6/2548	−4.82	#N/A	−2.49
	i3_LQ_LWC_c22293/f1p0/3430	−4.55	−2.31	−2.37
	i4_LQ_LWC_c9627/f1p4/4666	#N/A	−2.81	−4.93
CHI	i1_LQ_LWC_c26701/f1p0/1083	−2.10	−2.32	−4.31
	i1_LQ_LWC_c42238/f1p0/1048	#N/A	#N/A	−1.94
	i2_LQ_LWC_c66390/f1p2/2066	#N/A	#N/A	−1.63
F3′H	i2_LQ_LWC_c5478/f1p14/2479	2.31	#N/A	2.41
	i3_LQ_LWC_c10106/f1p9/3646	1.63	#N/A	2.09
	i1_LQ_LWC_c5071/f1p0/1485	−6.05	−7.62	−6.14
CYP75B1	i1_HQ_LWC_c27509/f19p0/1855	−4.80	−3.29	−6.41
	i1_HQ_LWC_c39849/f7p0/1891	#N/A	#N/A	−3.19
	i3_HQ_LWC_c29407/f2p0/3044	−2.91	−2.82	−3.15
	i1_LQ_LWC_c32858/f1p1/1996	−2.45	−1.75	−4.30
	i2_LQ_LWC_c47777/f1p4/2093	−4.79	−4.36	−5.65
HCT	i1_HQ_LWC_c29742/f2p0/1676	−2.27	−2.90	−1.95
	i1_HQ_LWC_c18545/f2p0/1773	#N/A	#N/A	−2.99
	i1_LQ_LWC_c17207/f1p1/1590	#N/A	#N/A	−5.13
	i1_LQ_LWC_c19818/f1p0/1549	#N/A	#N/A	1.45
	i1_LQ_LWC_c29534/f4p0/1653	#N/A	−1.69	−1.64
caffeoyl-CoA O-methyltransferase	i1_LQ_LWC_c38858/f1p0/1286	#N/A	#N/A	−1.59

## Data Availability

The datasets supporting the conclusions of this article are included within the article (and its additional files). Sequencing database for pearl millet could download from NCBI under the accession number PRJNA756390, and the data will be shared on reasonable request of the corresponding author.

## Supplementary Materials

The following are available online at https://www.mdpi.com/article/10.3390/genes12111716/s1, Figure S1. Pearl millet after 1, 3 and 7 h of heat treatment and control treatment. Figure S2. Correlation between samples. Figure S3. Changes of peroxidation scavenging enzyme activity of pearl millet under heat stress. (a) Changes of SOD activity in pearl millet under heat stress. (b) Changes of APX activity in pearl millet under heat stress. * represents *p* < 0.05, **** represents *p* < 0.0001. Table S1. Overview of the sequencing results of 18 samples. Table S2. The expression and functional annotation of DEGs in pearl millet after 1 h, 48 h and 96 h heat stress. Table S3. GO enrichment of pearl millet after 1 h, 48 h and 96 h heat stress. Table S4. KEGG enrichment of pearl millet after 1 h, 48 h and 96 h heat stress. Table S5. GO enrichment analysis after K-means clustering. Table S6. KEGG enrichment analysis after K-means clustering.

## Abbreviations

DEG	Differentially expressed gene
GO	Gene Ontology
KEGG	Kyoto Encyclopedia of Genes and Genome
HSP	heat shock proteins
ROS	reactive oxygen species
HSFS	heat stress transcription factors
SOD	Superoxide Dismutas
CAT	catalase
APX	ascorbate peroxidase
